# Histone Acetylation Modifiers in the Pathogenesis of Alzheimer’s Disease

**DOI:** 10.3389/fncel.2015.00226

**Published:** 2015-06-16

**Authors:** Xi Lu, Li Wang, Caijia Yu, Daohai Yu, Gang Yu

**Affiliations:** ^1^Department of Neurology, The First Affiliated Hospital of Chongqing Medical University, Chongqing, China; ^2^Department of Biotherapy and Hemato-oncology, Chongqing Cancer Institute, Chongqing, China; ^3^The Commonwealth Medical College, Scranton, PA, USA; ^4^Department of Clinical Sciences, Temple Clinical Research Institute, Temple University School of Medicine, Philadelphia, PA, USA

**Keywords:** Alzheimer’s disease, histone acetylation, histone deacetylase, histone deacetylase inhibitor, histone acetylase

## Abstract

It is becoming more evident that histone acetylation, as one of the epigenetic modifications or markers, plays a key role in the etiology of Alzheimer’s disease (AD). Histone acetylases and histone deacetylases (HDACs) are the well-known covalent enzymes that modify the reversible acetylation of lysine residues in histone amino-terminal domains. In AD, however, the roles of these enzymes are controversial. Some recent studies indicate that HDAC inhibitors are neuroprotective by regulating memory and synaptic dysfunctions in cellular and animal models of AD; while on the other hand, increase of histone acetylation have been implicated in AD pathology. In this review, we focus on the recent advances on the roles of histone acetylation covalent enzymes in AD and discuss how targeting these enzymes can ultimately lead to therapeutic approaches for treating AD.

## Introduction to Alzheimer’s Disease

Alzheimer’s disease (AD) is well known for the symptom of progressive memory loss. It is the most common neurodegenerative disease in the elderly, with an estimated 10–30% prevalence by age 85 or older and a 6–8% incidence in the same age group (Mayeux, 2003). Pathologically, AD is characterized by amyloid plaques and neurofibrillary tangles in select brain regions, including the temporal and parietal lobes, and in restricted regions within the frontal cortex and cingulate gyrus (Mattson, 2004; Tanzi and Bertram, 2005). The amyloid plaques are extracellular deposits of neurotoxic amyloid-β (Aβ) 40–42 peptides, highly involved in synaptic dysfunction and neuron death. Neurofibrillary tangles are intracellular accumulations of hyperphosphorylated tau, a microtubule-associated protein involved in the promotion and stabilization of microtubules (Ballatore et al., 2007). The “plaques” and “tangles” play a critical role in the oxidative stress damage, energy metabolism disturbance, and abnormal cellular calcium homeostasis, and hence increase the risk for developing AD (Mattson, 2004).

There are more and more genetic factors found to be strongly implicated for causing or increasing the risk of AD development. In the identification of familial AD (FAD) genes, mutations in the presenilin 1 (PS1) gene are the most frequent (81%), followed by amyloid precursor protein (APP) (14%), making PS1 variation as the well-known genetic cause of FAD (http://www.molgen.ua.ac.be/ADMutations). PS1 is the catalytic core component of γ-secretase, one of the sequential cleavage enzymes to APP, and mainly produces the neurotoxic Aβ peptides that are responsible for neuronal degeneration and cognitive dysfunction in AD (Scheuner et al., 1996). In clinics, the majority of AD cases are sporadic without any linkage to family history. The apolipoprotein E (APOE) ε4 allele has been linked to high susceptibility to developing sporadic AD (SAD), and the presence of an APOE ε4 allele is also associated with amyloidogenic processing and neurofibrillary tangles (Roses, 1997).

The complex pathogenetic background of AD implies that both genetic and non-genetic factors are possible causes in the diverse signaling pathways leading to AD. There is a multitude of environmental factors, for example, physical exercise, diet, cognitive training, education level, and head trauma, that may play a role in the development of AD. However, the research regarding environment and gene-environment interactions has not matured enough to yield a clear picture about AD (Young et al., 1999; Cotman and Berchtold, 2002; Mattson, 2003; Mayeux, 2003; Ngandu et al., 2015).

## Histone and Non-Histone Acetylation and Alzheimer’s Disease

**Figure 1:**
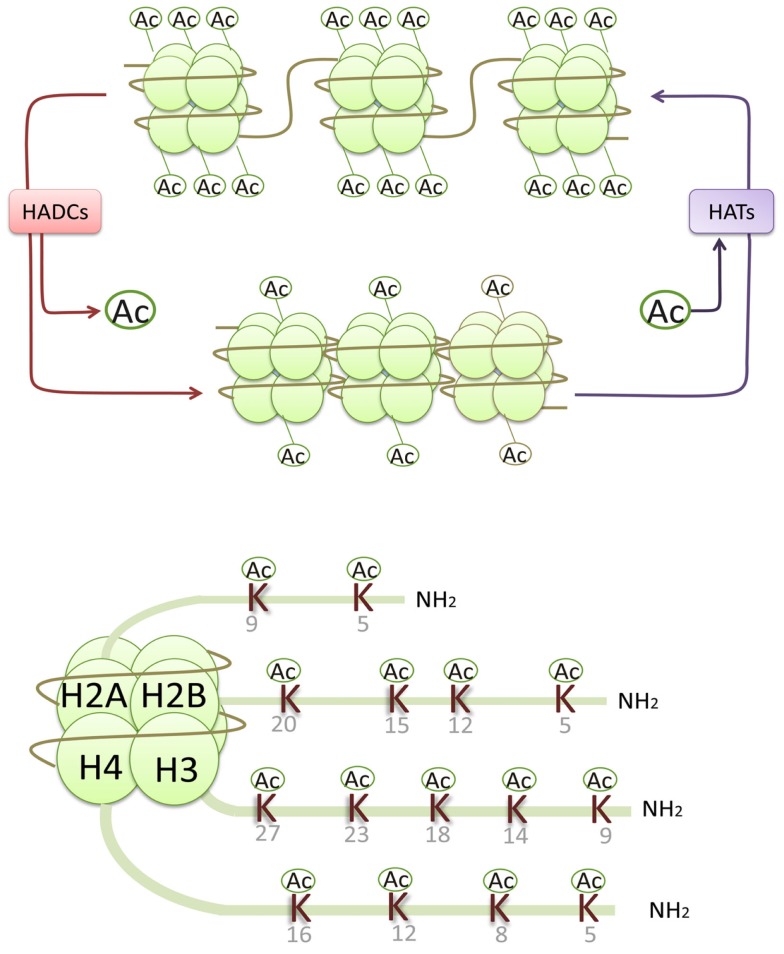
**Histone acetylases (HATs) reversibly transfer the acetyl groups to the core histones by neutralizing the positive charge of the lysine (K) residues in histone N-terminals, resulting in an open chromatin structure accessible to transcriptional factors and an activation of gene transcription, while histone deacetylation usually leads to gene transcriptional repression**.

Epigenetic processes integrate abundant signals of genetics and environment into phenotypic outcomes. They are considered heritable alterations in gene expression without any changes in their coding sequence (Egger et al., 2004). Such epigenetic processes include histone modifications (acetylation, phosphorylation, methylation, ubiquitination, ADP ribosylation, and sumoylation), DNA methylation, and non-coding RNAs (Goldberg et al., 2007). Histone acetylation and deacetylation regulate gene transcription by altering the chromatin structure and the accessibility to transcription factors. The alkaline histones are abundantly found in eukaryotic cell nuclei and they are chief protein components of chromatin. They form octamers that wrap around DNA to form nucleosomes, and each histone octamer consists of two distinct copies of the four core histones (H2A, H2B, H3, and H4). Histone H3 and H4 acetylation have been demonstrated to be markers of an “open” configuration of chromatin. Core histones are acetylated on lysine residues at the N-terminal tails and facilitate gene transcription by neutralizing the positive charge of histone tails and reducing the binding of histone to negatively charged DNA (Kouzarides, 2007). In general, histone acetylation is closely associated with gene transcriptional activation, while histone deacetylation usually leads to gene transcriptional repression (Figure 1).

Dysregulation of histone acetylation is involved in a variety of signal transduction pathways such as cell differentiation, cell apoptosis, vascular remodeling, inflammation reaction, immune responses, neuronal plasticity, and metabolic reprograming (Powell et al., 1999; Barnes, 2009; Keenen and de la Serna, 2009; Pons et al., 2009; Witt et al., 2009; Graff et al., 2012; Mihaylova and Shaw, 2013). Altered acetylation of both nuclear and cytoplasmic non-histone proteins has also been associated with AD, including NF-κB (Chen et al., 2001), p53 (Barlev et al., 2001), alpha tubulin (Perez et al., 2009), and tau (Min et al., 2010; Irwin et al., 2012), adding another level of regulation to molecular pathways in AD.

Acetylases p300/CBP acetylate multiple lysine residues of NF-κB, including Lys-122, -123, -218, -221, and -310 (Chen et al., 2001). Park et al. found that PCAF (p300/CBP associated factor) selectively acetylates the Aβ-induced activation of NF-κB at Lys-122 by the Western blot using an antibody against acetyl-NF-κB (K122), while on the other hand, the PCAF inhibitor C-30-27 selectively inhibits acetylation-dependent NF-κB at Lys-122 and suppresses the NF-κB-mediated inflammatory response induced by Aβ in both BV2 and Neuro-2A cells (Park et al., 2013, 2015). However, HDAC inhibitor valproic acid (VPA) also decreases the mRNA levels of NF-κB in both plasma and hippocampus of Tg6799 AD mice (Noh and Seo, 2014). These discordant results suggest that the acetylation-induced regulation of NF-κB expression in AD pathology needs more clarification and research. Furthermore, NF-κB is also tightly regulated by another deacetylase sirtuin SIRT1 in AD *in vitro* models (Chen et al., 2005; Marwarha et al., 2014).

The tumor suppressor and transcription factor p53 is modified by acetylation, which does not increase DNA binding of p53 but promotes coactivator recruitment and histone acetylation (Barlev et al., 2001). One research team found that acetylations of p53 are significantly increased in AD brain tissue, and p300 activities might converge to increase p53 levels in AD brains by inducing p53 acetylation in its C-terminal domain (Aubry et al., 2015). It has also been suggested that CBP/p300 induced p53 hyperacetylation is enriched during neuronal outgrowth and maturation (Tedeschi et al., 2009). For the HDAC, SIRT1 agonist resveratrol decreases the acetylation of p53 and hence rescues increased p53 acetylation in the CK-p25 model of neurodegeneration (Kim et al., 2007).

It is known that tau is acetylated in neurodegeneration and that tau acetylation suppresses degradation of phosphorylated tau (p-tau). Histone acetyltransferase p300 regulates the tau acetylation and the deacetylase SIRT1 mediates the tau deacetylation (Min et al., 2010). Irwin et al. also observed the acetylated-tau pathology in a spatial distribution pattern similar to hyperphosphorylated-tau. They detected the acetylated-tau at lysine 280 in AD and showed that acetylated-tau pathology is largely intracellular and present throughout all stages of AD progress, especially moderate- to severe-stage cases. The acetylated-tau may contribute to tau-mediated neurodegeneration by reducing solubility and microtubule assembly and increasing tau fibrillization (Irwin et al., 2012). CREB-binding protein (CBP) acetylates tau at Lys280 within the tau microtubule-binding motif, and that acetylation of tau possibly leads to increased tau aggregation (Cohen et al., 2011). Tau acetylation also correlates with the concentration of p300; however, p300 and CBP may preferentially acetylate different residues in tau, thus differentially affecting tau’s intrinsic propensity to aggregate (Cook et al., 2014a,b). Reversely, HDAC6 activity presumably enhances the deacetylation of both tubulin and tau, which may contribute to tau-microtubule interactions and microtubule stability (Cook et al., 2014a,b).

The discovery of the association of the impairment of histone acetylation homeostasis with the memory deficit during the past 10 years brought about a rapid increase in the knowledge of cognitive dysfunction of neurodegenerative disorders. Acetylation of the hippocampal histones (H2B, H3, and H4) are transiently increased in normal mice during learning processes, suggesting that histone acetylation is essential for memory consolidation (Levenson et al., 2004; Levenson and Sweatt, 2005; Fischer et al., 2007; Koshibu et al., 2009; Peleg et al., 2010). Gjoneska et al. not only found decreased H3K27 acetylation at regulatory regions of synaptic plasticity genes in the p25 transgenic model of AD but also found increased H3K27 acetylation at regulatory regions of immune response genes. These changes in histone acetylation correspond to the changes in transcription (Gjoneska et al., 2015). While histone acetylation shows an overall decrease in the aged mice, the application of HDAC inhibitors reverses such decreases in the global histone acetylation and improves the memory deficits *in vivo* (Chuang et al., 2009; Graff et al., 2012; Walker et al., 2012). However, some studies found that histones are hyperacetylated in neuroblastoma cells by Aβ peptide deposits (Guo et al., 2011; Gu et al., 2013; Lu et al., 2014). It is reported that some APP metabolism related genes are also regulated by histone acetylation. In our previous studies, we found that H3 in the promoters of PS1 and BACE1, a β-secretase to APP for Aβ peptides, is hyperacetylated in N2a cells transfected with Swedish mutated APP (Lu et al., 2014). Similar H3 hyperacetylation of BACE1 promoters has been reported in APP/PS1/tau triple transgenic mice (Marques et al., 2012). Nevertheless, neprilysin (NEP), a major degrader of Aβ peptides, is downregulated due to the decreased H3 acetylation at the gene promoter regions in hypoxia stimulated mouse cortical and hippocampal neurons (Wang et al., 2011). These discordant results indicated that the role of histone acetylation abnormality in AD pathology is still unclear, and a thorough understanding of these issues would likely lead to the development of effective treatments for AD.

### Histone deacetylase and alzheimer’s disease

Generally, histone deacetylases (HDACs) repress transcription by removing an acetyl group from the histone tail and compacting chromatin. Mammalian HDAC enzymes are classified into four major categories in line with their homology to yeast: (1) Class I HDACs consist of HDACs 1, 2, 3, 8; (2) Class II HDACs further separate into two subclasses, class IIa including HDACs 4, 5, 7, 9 and class IIb HDACs 6, 10; (3) Class III HDACs are named as sirtuins and include SirT1-7, sharing their homology sequence to the yeast Sir2; and (4) Class IV HDACs include only HDAC11 (Chuang et al., 2009). Class I, II, and IV HDACs are zinc-dependent enzymes, while Class III HDACs enzymes are dependent on nicotinamide adenine dinucleotide (NAD+).

HDAC2 is widely expressed in the central nervous system and negatively regulates memory and synaptic plasticity. The hippocampi of HDAC2-overexpressing mice showed hypoacetylation of histone H4 on lysines 12 and 5 (H4K12, H4K5) but not H3K14, accompanied by decreased synapse number and synaptic plasticity, resulting in impaired memory formation. The mechanism is believed to be via the binding of HDAC2 to the promoters of synaptic-plasticity-related genes and thereby negatively regulates their transcriptions (Guan et al., 2009). HDAC2 expression is increased in the post-mortem brain samples of AD patients. Knocking down HDAC2 by short-hairpin-RNA restores the structural synaptic plasticity and memory impairments in CK-p25 mice by the way of HDAC2 reversely regulating H4K12 acetylation of the memory related gene promoters (Graff et al., 2012). These findings underline the important role of HDAC2-regulated chromatin modification in regulating the synaptic plasticity and memory formation in the cognitive impairment context of AD.

An increased HDAC6 protein level is observed in the cortices and hippocampi of AD postmortem brain samples (Ding et al., 2008), and reducing endogenous HDAC6 restores the learning and memory deficits and α-tubulin acetylation in an AD mouse model (Govindarajan et al., 2013). It is believed that cytoplasmic deacetylase HDAC6 participates in tau metabolism, one pivotal process involved in neurofibrillary tangles in AD (Ding et al., 2008). On one hand, HDAC6 levels positively correlate with the tau burden, and a decrease in HDAC6 activity or expression significantly prevents tau aggregation and accelerates tau clearance via acetylation of tau on KXGS motifs (Cook et al., 2012, 2014a,b). Conversely, tau can also function as an inhibitor of HDAC6, and overexpression of tau corresponds with tubulin hyperacetylation (Perez et al., 2009). On the other hand, an inhibitor of HDAC6-mediated α-tubulin deacetylation, tubacin represses tau phosphorylation at T231, a critical tau function site (Ding et al., 2008), and decreases cell motility without affecting the stability of microtubules (Haggarty et al., 2003). Moreover, inhibition of HDAC6 activity also blocks mitochondrial lengthening and transportation in Aβ-induced hippocampal neurons, perhaps in a GSK3β-dependent manner (Chen et al., 2010; Kim et al., 2012).

Sirtuins are well known for their association with human longevity and their potential ability to delay the onset of age-related AD. AD patients have shown a significant reduction of SIRT1 expression in the parietal cortex, but not in the cerebellum, which is at par with their severity of cognitive impairment. Further analyses confirm that the cortical SIRT1 loss is negatively associated with the accumulation of Aβ and tau. However, the reduction of SIRT1 is not found in APP/PS1/tau triple-transgenic animals (Julien et al., 2009). In p25 transgenic mouse, another model of AD, increasing SIRT1 activity reduces neurodegeneration in the hippocampus. SIRT1 agonist resveratrol decreases the acetylation of the SIRT1 substrates PGC-1α and p53 and prevents the learning impairment (Kim et al., 2007). Meanwhile, SIRT1 mediates synaptic plasticity and memory formation via modulation of miR-134 expression (Gao et al., 2010). Sirtuin SIRT1 also tightly regulates NF-κB activity in AD progression. Chen et al. discovered that SIRT1 reduces Aβ-induced toxicity by inhibiting NF-κB signaling, and SIRT1 overexpression suppresses the Aβ-dependent NF-κB activation, blocking the neuropathogenic inflammatory reaction in primary neurons (Chen et al., 2005). On the other hand, inhibition of SIRT1 induces an increase of NF-kB reporter activity and BACE1 promoter activity, but SIRT1 inhibitor sirtinol does not change the binding of NF-κB in the BACE1 promoter region (Marwarha et al., 2014). Far-ranging functions of SIRT1 have been explored in the pathogenesis of aging and neurodegeneration, raising the possibility for its use in therapeutic interventions for AD.

### HDAC inhibitors

The molecular and clinical implications of the HDAC inhibitors were initially identified in cancer therapy (Carew et al., 2008). Notably, the HDAC inhibitors restores the learning and memory deficits in AD mouse models, especially in the early stage AD mouse cortex, making it a promising drug for AD (Fischer et al., 2007; Vadnal et al., 2012). In fear-conditioned AD mouse models, the HDAC inhibitors such as trichostatin A (TSA), VPA, SAHA (vorinostat) or sodium butyrate increase the synapse remodeling and enhance the contextual memory by regulating H3/H4 acetylation of relevant gene promoters to enhance hippocampal long-term potentiation (Fischer et al., 2007, 2010; Francis et al., 2009; Guan et al., 2009; Kilgore et al., 2010; Ricobaraza et al., 2012). At the molecular level, sodium butyrate recruits acetylated H3/H4 to the DHCR24 enhancer and increases gene expression which is reduced in the temporal cortex of an AD patient’s brain (Drzewinska et al., 2011).

Nevertheless, most of the HDAC inhibitors are non-selective and target not only nuclear histones but also cytoplasmic non-histone proteins. Some HDAC inhibitors such as TSA selectively regulate the expression of memory-related genes rather than globally alter gene expression in a non-specific manner (Vecsey et al., 2007). It is hence expected that certain structural features of the HDAC inhibitors are identified with conferring potency and specificity (Haggarty et al., 2004). Khan et al. compared the clinically relevant HDAC inhibitors against the rhHDAC (recombinant human HDAC) microforms and identified the potency and selectivity of ten microforms that increase histone acetylation in Hela cells (Khan et al., 2008). The microforms of these four groups of HDACs and main HDAC inhibitors are listed in Table 1.

**Table 1:** **HDAC family and HDAC inhibitors**.

HDAC subclass		Localization	Selective-inhibitor	Non-selective-inhibitor
Class I zinc-dependent	HDAC1	Nucleus	MS-275, FK-228	TSA, VPA
	HDAC2	Nucleus	FK-228, Apician	Butyrate, SAHA
	HDAC3	Nucleus, cytoplasm	Apician, RGFP136	MGCD0103
	HDAC8	Nucleus, cytoplasm		
Class Iia zinc-dependent	HDAC4	Nucleus, cytoplasm		TSA, phenyl butyrate
	HDAC5	Nucleus, cytoplasm		
	HDAC7	Nucleus, cytoplasm		
	HDAC9	Nucleus, cytoplasm		
Class Iib zinc-dependent	HDAC6	Cytoplasm	Tubacin	TSA, SAHA
	HDAC10	Nucleus, cytoplasm		
Class III NAD^+^-dependent	SirT1	Nucleus	Suramin	Nicotinamide
	SirT2	Nucleus, cytoplasm	Suramin, AGK2	
	SirT3	Mitochondria		
	SirT4	Mitochondria		
	SirT5	Mitochondria		
	SirT6	Nucleus		
	SirT7	Nucleus		
Class IV zinc-dependent	HDAC11	Nucleus

The HDAC inhibitors regulate specific gene transcription not only by acetylating the histones of the gene promoter or enhancer directly but also by acetylating the genes relevant to the transcriptional regulator such as Sp1 and HNF4a indirectly (Ryu et al., 2003; Yang et al., 2009). Still, only 8–20% of all the genes analyzed display permissive conformations for transcription in cells with the HDAC inhibitors treatment. The HDAC inhibitors are likely to modulate transcriptional programs of certain relatively specific genes (Fischer et al., 2007).

### Histone acetylases and alzheimer’s disease

Histone acetylases (HATs), contrary to HDACs, are recruited to reversibly transfer acetyl groups to lysine residues of histone N-terminals, resulting in an open chromatin structure accessible to transcriptional factors. HATs are broadly divided into two main groups according to their cellular localization, the nuclear A-type HATs, and the cytoplasmic B-type HATs. A-type HATs share a highly conserved motif, including an Acetyl-CoA binding site, and mainly acetylate the histones of nuclear chromatin, which directly impacts activation of transcriptional responses. Conversely, B-type HATs acetylate the newly synthesized cytoplasmic histones, making ample histones free to enter the nucleus. A-type group contains three primary subclasses of HATs based on structural homology in their primary sequences: (1) GNAT (Gcn5-related N-acetylases) family, containing human Gnc5, PCAF, and elongator complex protein 3(ELP3); (2) MYST family, comprised of Tip60, MOZ/MYST3, MORF/MYST4, HBO1/MYST2, and HMOF/MYST1; and (3) p300/CBP family, represented by p300 and CBP (Roth et al., 2001). The groups of the HAT families are represented in Table 2.

**Table 2:** **HAT family**.

HAT subclass (type A)		Histone substrate
GNAT family	Gnc5, PCAF, ELP3	H3K9, 14, 18, 36
MYST family	Tip60, MOZ, MORF, HBO1, HMOF	H4K5, 8, 12, 16, H3K14
p300/CBP family	p300, CBP	H2AK5, H2BK12,15, H3K14, 18, H4K5, 8
Transcription factor related	TFIIIC, TAF1	H3K9, 14, 18
Nuclear receptor co-activators	SRC, ACTR, P160, CLOCK	H3/4

Among the studies of HATs and their associations with AD development, Tip60 is most generally believed to be a valid therapeutic approach to slowing down or even stopping the dementia progression. Tip60 was initially discovered through its interaction with the HIV-1 transactivator protein Tat. Lorbeck et al. found that the HAT activity of Tip60 regulates neuronal-specific gene profiles that are linked to the behavior, learning, and memory in Drosophila (Lorbeck et al., 2011). This group further proved that the activity of Tip60 controls the synaptic remodeling and structure in the Drosophila neuromuscular junction (Sarthi and Elefant, 2011). They also pointed out that in the APP transgenic Drosophila, Tip60 HAT activity loss increases the APP transcriptional expression, which induces lethality and a neuronal apoptotic reaction (Pirooznia et al., 2012). These results suggest a key neuroprotective role of Tip60 HAT activity for AD neurodegenerative pathology in humans.

It was implicated that the loss of HAT activity in CBP/p300 is related to the neuronal survival and long-term memory. The loss or over-expression of CBP/p300 are both responsible for the neuronal death, implying that only a fine-tuning of CBP HAT activity is neuroprotective in the context of AD development (Rouaux et al., 2003). Behavior-trained rats display an increase in the activity/expression of CBP/p300 and PCAF, accompanied by hyperacetylated H2B/H4 in the promoters of the synaptic-plasticity-related genes (Bousiges et al., 2010). Meanwhile, CBP-dependent transcriptional neuroadaptation is required for environmental enrichment-induced neurogenesis and cognitive enhancement (Lopez-Atalaya et al., 2011). AD pathological contexts also show a critical CBP/p300 loss with histone H3 deacetylation (Rouaux et al., 2003). Furthermore, CBP/p300 inhibitor EID1 has been successfully utilized to identify the protective role of CBP/p300 in the memory processes. Increase of EID1 nuclear translocation induces a decline in the spatial learning and long-term potentiation in the hippocampus by reducing the neuronal structural protein βIII-tubulin expression (Liu et al., 2012), whereas the elimination of CBP does not affect the neuronal viability (Valor et al., 2011).

Nonetheless, the p300 acetyltransferase activity is upregulated and may interact with the hyperacetylation of tau in AD brains (Aubry et al., 2015). In the FAD pathology context, CBP/p300 shows a negative role. PS1 mutations repress the proteasomal degradation of CBP/p300 and up-regulate the CREB-mediated gene transcription in murine neurons (Marambaud et al., 2003). In addition, PS1/2 knock-out mice reveal a decline in the CBP/p300 expression (Saura et al., 2004). In our previous study, we also found that the Swedish APP mutation induces the p300 expression up-regulation in N2a cells (Lu et al., 2014). These ambiguous results point to the need for further studies on the HAT activity of epigenetic CBP/p300 functions in the development of AD.

Some studies point out that enhancement of HAT activity, similar to the HDAC inhibitors in function, is another potential therapeutic approach for AD (Rouaux et al., 2003; Tedeschi et al., 2009; Bousiges et al., 2010; Liu et al., 2012; Park et al., 2013, 2015). Compared to the VPA treatment, HAT activators CSP–CTPB injections potently induce the histone H2B acetylatation in the hippocampus (Selvi et al., 2010). The CREB-CBP transcriptional complex is also involved in the HDAC inhibitors treatment, inducing the enhancement of the hippocampus-dependent memory and synaptic plasticity (Vecsey et al., 2007).

## Therapeutic Perspectives

Accumulating evidences *in vivo* and *in vitro* support the contention that histone modification and dysfunction are associated with the etiology of AD. Experimental evidence suggests that HDAC inhibitors treatments, both ameliorate cognitive deficiencies, protect against memory impairment, promoting the possibility of their further development in clinical trials in AD patients. However, several intractable and complex issues remain and need to be addressed for further experimental and clinical applications. One of the most urgent issues is to effectively identify the isoform-specificity of the HDAC inhibitors, as non-selective HDAC inhibitors lead to a loss in the signal-non-specific responses. Despite animal experiments, pharmaceutical and clinical characteristics of an HDAC inhibitor treatment such as brain permeability, efficacy, toxicity, and distribution need to be further investigated in future randomized placebo-controlled clinical trials with a PK-PD and/or imaging component.

Although the protective role of the HDAC inhibitors in AD is widely known, the pattern of histone acetylation in the AD pathology is still not well understood. While the global histone acetylation is decreased in some AD animal and cellular models, the histone acetylation is either increased or decreased in some specific regions of certain genes that function disjointedly or in agreement. For example, the memory-related genes have histone hypoacetylation, resulting in low expression of these genes (Levenson et al., 2004; Levenson and Sweatt, 2005; Fischer et al., 2007; Koshibu et al., 2009; Peleg et al., 2010). In our previous study, we found that the genes directly related to AD pathology such as PS1 and BACE1 are hyperacetylated, resulting in the high transcription of these genes (Lu et al., 2014). The global histone acetylation may be a result of varying degrees of histone acetylation modulation of various genes. Furthermore, it appears that not all histones impact memory formation. It is interesting to note that training or environmental enrichment can induce the acetylation of some specific histones. For example, it was observed that acetylation of histone H3 and H4 are increased in the hippocampus and cortex of p25 transgenic mice following environmental enrichment (Fischer et al., 2007). During learning, a specific deregulation of the histone H4 lysine 12 acetylation is observed in aged mice (Peleg et al., 2010). What is more, CBP/p300 might affect the acetylation of specific histones/non-histone proteins in neurodegenerative disease. Elimination of CBP in the forebrain principal neurons preferentially reduced acetylation of histone H2A and H2B in Rubinstein-Taybi syndrome (Valor et al., 2011). Increased amounts of EID1, an inhibitor of CBP/p300, in hippocampal neurons significantly reduced the acetylation of H3 and p53, but not other histones (Liu et al., 2012). Moreover, it is possible that the overall level of histone acetylation depends on different brain regions, animal models, cellular type, and gene regions (Graff and Mansuy, 2009). From this point of view, it is important to undertake a more global view to look at the mechanism of epigenetic modulation as a therapeutic target in AD. The chromatin modification of the AD-related genes needs further investigation in AD patients and *in vivo* or *in vitro*.

Nonetheless, there is no doubt that HDAC inhibition presents a novel promising avenue for the development of therapeutic strategies for Alzheimer disease and its associated learning and memory impairments. New insights of histone acetylation in the etiology of AD are awaiting further exploration, making previously irreversible brain disorders potentially reversible.

## Conflict of Interest Statement

The authors declare that the research was conducted in the absence of any commercial or financial relationships that could be construed as a potential conflict of interest.
